# Supplementary figures and images for: Bacterial communities of Aedes aegypti mosquitoes differ between crop and midgut tissues
Source: PLoS Negl Trop Dis. 2023 Mar 29;17(3):e0011218. doi: 10.1371/journal.pntd.0011218 (PMC10085046; doi:10.1371/journal.pntd.0011218)

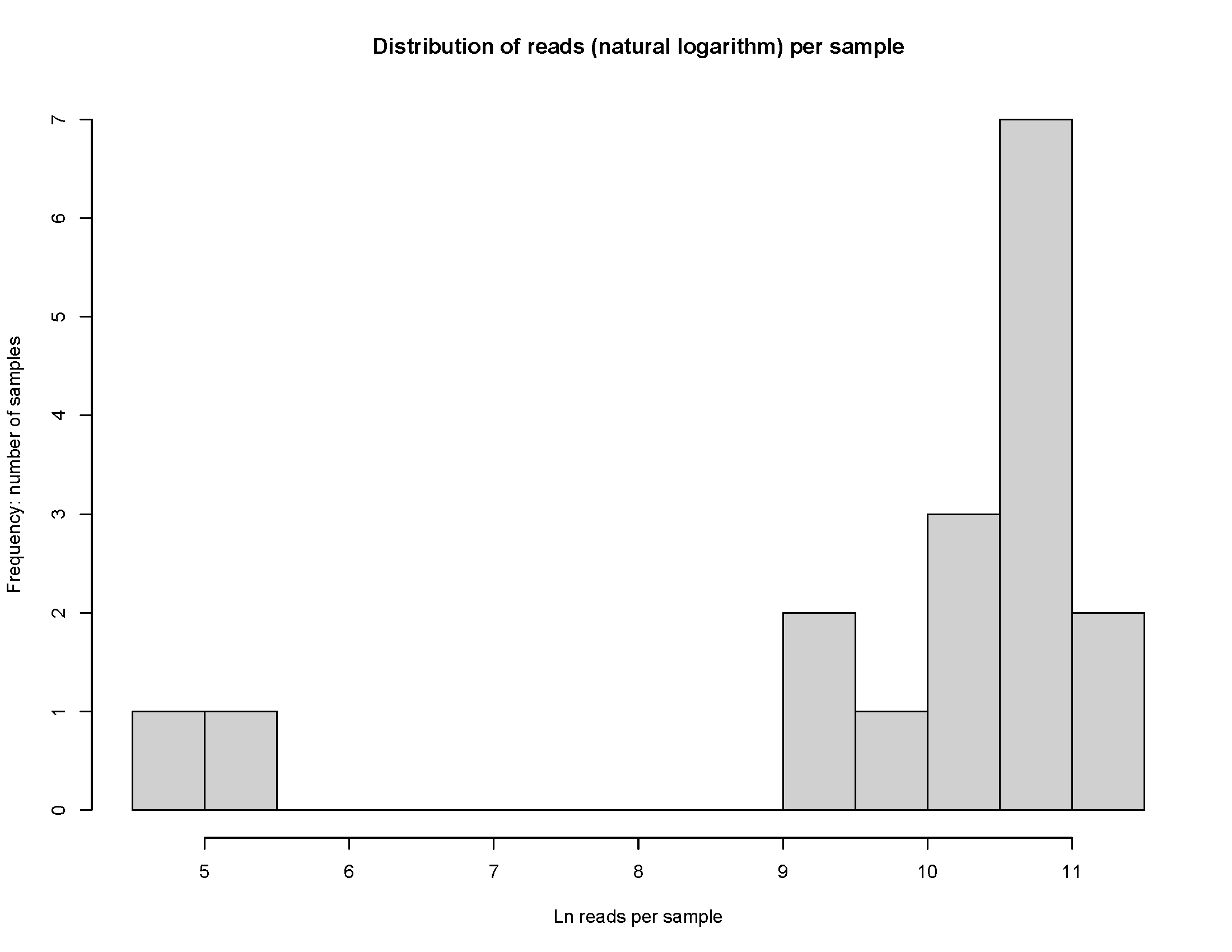

Supplement: S1 Fig — (TIF) [file pntd.0011218.s001.tif]

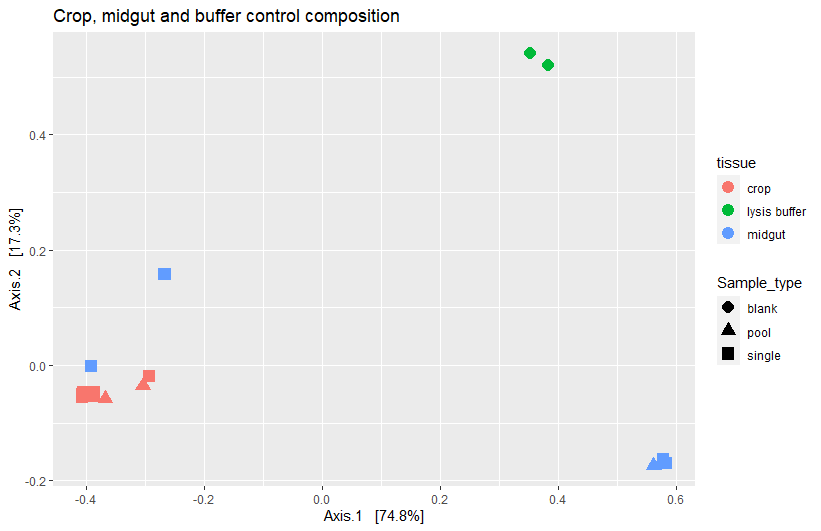

Supplement: S2 Fig — PCoA using Bray Curtis dissimilarity values shows that buffer blanks (shown in green) group together and separately from experimental samples. (TIF) [file pntd.0011218.s002.tif]

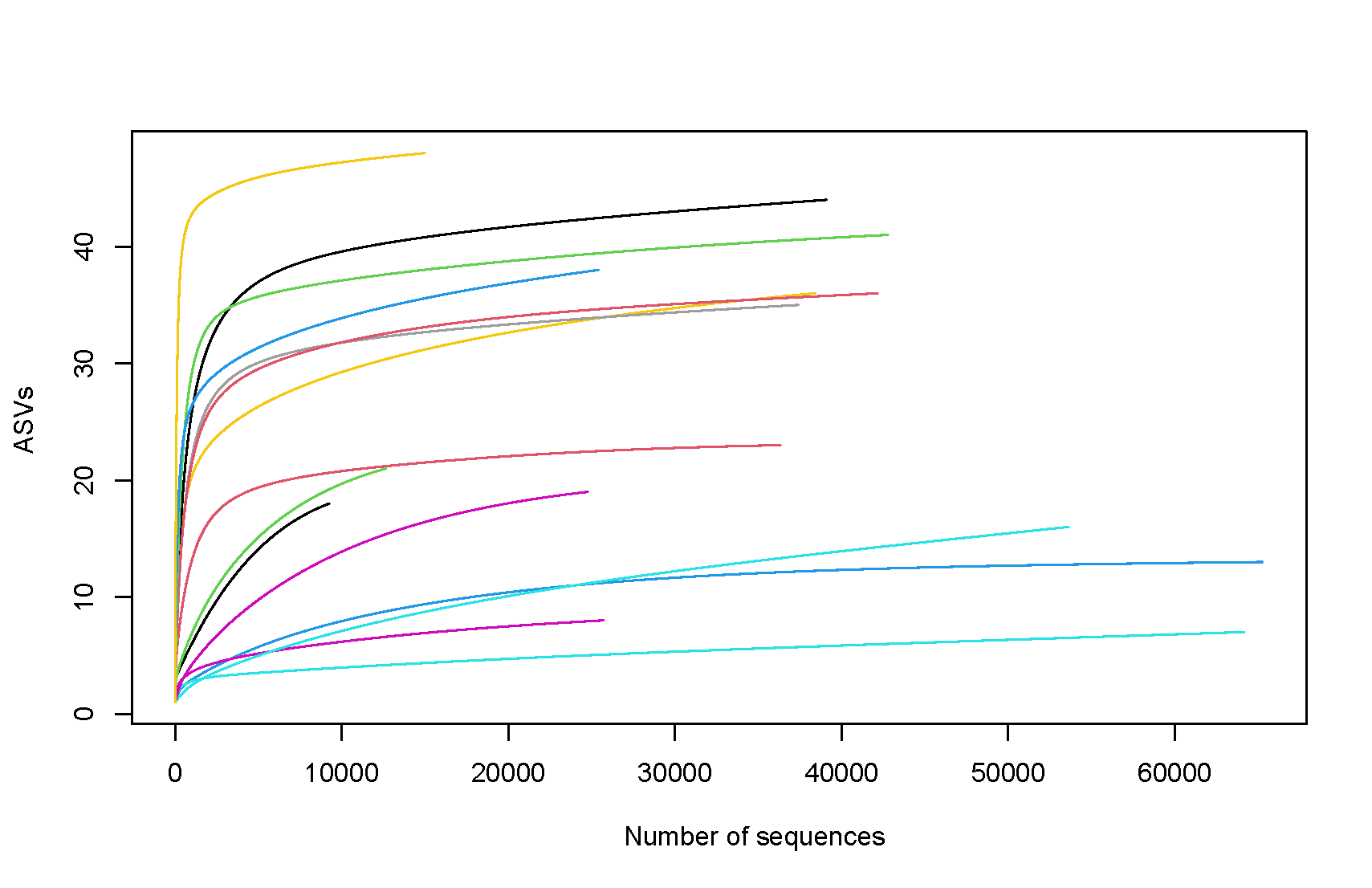

Supplement: S3 Fig — (TIF) [file pntd.0011218.s003.tif]
