# Supplementary material for: Bacterial communities of Aedes aegypti mosquitoes differ between crop and midgut tissues
Source: PLoS Negl Trop Dis. 2023 Mar 29;17(3):e0011218. doi: 10.1371/journal.pntd.0011218 (PMC10085046; doi:10.1371/journal.pntd.0011218)
Supplement: Attachment — Submitted filename: Comments.docx [file pntd.0011218.s006.docx]

**General comments**

Bacterial communities within the mosquito body play important roles in host health and some of them could be harnessed as novel tools for mosquito control. Understanding the composition and structure of these microbiota in different mosquito tissues is therefore an important aspect of mosquito biology. This research uses a laboratory colony of Aedes aegypti to characterize and compare the bacterial communities in the crop and midgut tissues using both culture-dependent and culture independent techniques. As previously reported for lab mosquitoes, the authors reported low bacterial diversity in the two tissue types. They also found that the two tissue types host different bacterial taxa with the dominance of Weeksellaceae in the midgut samples and Acetobacteraceae in crop samples. Some differences in bacterial composition were also reported between pooled and individual samples especially for the midgut samples. The research is well thought out and the manuscript is well written. My only disappointments are that the authors: 1) used a laboratory colony reared using artificial diet which does not reflect they dynamics we would expect in nature, 2) the studies were conducted using 4-6 days old sugar-fed mosquitoes. It would have been great to include newly emerged mosquitoes as sugar-feeding probably facilitated the transfer of bacteria between tissues. Comparing newly emerged adults’ vs sugar-fed adults could have made it possible to determine whether there was any bacteria transfer between tissues after sugar feeding. I also felt that the sample size was too small especially for individually processed mosquitoes and wondered why authors decided to pool the mosquitoes instead of processing them individually given the well-documented inter-individual variation in microbial communities. Having said this, I believe the work presented here is a great contribution to the scientific community.

**Specific comments**

-No control of larval experiment

-No newly emerged mosquitoes

-Supplementary figure 1.I would like to see the read counts rather than the frequency. What does 4, 6, 8, 10 and 12 represent in the x axis? You imply it is reads per samples, does it mean that the samples had only 4, 6, 8, 10 and 12 reads?

-Supplementary figure 2: the shapes are so tiny, I can hardly see them. Please, make them larger.

-Line2 224- 225: “…then scaled reads per sample based on the smallest read count as suggested by [51] in order to preserve proportions.” What was the sequencing depth per sample? What was the smallest and largest read count? You can provide this information here or in the results (see later comments). This information is presented in the supplementary file but it would be good to have it in the results section.

-Alpha diversity. I would like to see chao1 values and Shannon diversity index or a justification on why they are not reported.

-Figure legends: is it necessary to include all this information when it is already stated in the text?

-Please include data on the sequencing depth per sample (include minimum and maximum values, mean and SD or SE).

- I would also like to see the rarefaction curves to know whether the samples reached saturation or not.

-Please, provide a figure showing the composition of different bacterial phyla and genera in crop and midgut samples. Any reason why only the family level classification is presented?

-Figure 3: how can you explain the huge difference between pooled and single midgut samples? Could it have been contamination from the crop? How certain are you that is was transfer from the crop?

Lines 363-371: I would like to see results of Shannon diversity index (which accounts for species abundance) and Chao1.

-Figure 3: Please, increase the size of the shapes. They are too small.

- Lines 395-397: “The communities were dominated by reads that map to the genera *Elizabethkingia* (Family: Weeksellaceae), *Tanticharoenia* (Family: Acetobacteraceae), and/or *Asaia* (Family: Acetobacteraceae)”. Figure 3 presents data at family level. Please, present the results at genus level.

-Lines 448-449: “….five functional categories were only found in midguts, including

449 manganese oxidation.” Please, list the other four, they are not easy to spot from the figure. It would also be good to list the 15 that were shared.
