# Supplementary material for: Bacterial communities of Aedes aegypti mosquitoes differ between crop and midgut tissues
Source: PLoS Negl Trop Dis. 2023 Mar 29;17(3):e0011218. doi: 10.1371/journal.pntd.0011218 (PMC10085046; doi:10.1371/journal.pntd.0011218)
Supplement: Attachment — Submitted filename: 20221222_ResponetoReviewers.docx [file pntd.0011218.s007.docx]

We thank both reviewers for their thoughtful and helpful comments. We have addressed each comment in red below.

**Reviewer #1**

Reviewer #1: When studying potential impact of microbiota in mosquitoes’ biological function, it may be more relevant to investigate the microbiota composition of individual mosquitoes. Please provide rationale for using pooled vs individual samples.

The reviewer is correct when stating that individual samples can be more relevant when investigating the functional profiles of bacterial communities. It has been observed with beta diversity in several models that pooling may remove interindividual variation which is principally driven by rare taxa or unique life history traits. Nonetheless, pooling remains a viable technical alternative when samples are low yield, or to describe broad trends revealed by the community structures. In our study, we were uncertain how many reads we would be able to isolate from crop tissues and were concerned that using only individual samples would yield unusable results. Therefore, we chose a combined approach of sampling individuals and pools. This had the benefit of ensuring we would obtain usable data and allowed us to evaluate whether both single and pooled samples revealed similar clustering patterns based on our hypothesis of tissue driven beta diversity differences. We found that, while single samples revealed more inter-individual variation, beta diversity trends were largely preserved whether we analyzed single or pooled samples. Given the scarcity of community functional predictions from *Ae. aegypti* in general and from tissue-specific organs especially, we felt this analysis would provide important insight to the larger community. We added the following statements:

In lines 178-180 in the methods section “We were uncertain how many bacteria are present in crops and thought it likely these tissues would have low biomass. To increase the likelihood of successful community profiling, we chose to analyze individuals and pools for high throughput sequencing.”

in lines 510-513 in the discussion “This suggests that analyzing single rather than pooled samples can be critical to assess inter-individual variation. Microbial communities differed significantly in composition and structure between tissues (beta diversity), however whether the samples were single or pooled was not a significant predictor of beta diversity.”

“Consistently with other models [102,103], we observed that the functional trait abundances of the bacterial communities reflect the overall beta diversity, as samples cluster predominantly in a by-tissue pattern (Figure 8)”.

Provide rationale for the use of TSA and M9 media. Is there anticipation to see any differences in the types of colonies grown between these two media. If so, please include this in the discussion.

We chose to use two very different media types to increase the likelihood of culturing as many bacterial strains as possible. We chose TSA and M9 because one (TSA) is a rich media with high nutrient content and the other (M9) is a minimal media with very low nutrient content. We took this approach because multiple studies have shown that rich media, while excellent for isolating a wide variety of bacteria, can favor fast-growing species and prevent isolation of rare or slow-growing species (Medina et al., 2017; Connon and Giovannoni, 2002; Vartoukian et al., 2010). We chose to supplement our use of TSA with a low nutrient media, and we chose M9 because it is completely defined. Using a defined media will allow for downstream applications such as further physiological characterization of the bacterial isolates.

We have added the following text to the materials and methods on lines 148-151 to explain this: “We chose TSA because it is a high-nutrient media commonly used to isolate diverse environmental bacteria. We also used a low-nutrient media (M9) because use of only a rich media can favor fast-growing species and prevent isolation of rare and/or slow-growing species (Medina et al., 2017; Connon and Giovannoni, 2002; Vartoukian et al., 2010).”

Reviewer #1: Was there any sequences of animal parasites or symbionts detected in the 16S sequencing? Metabolic profiles 10-20% heat map in Fig 8 seems to indicate, this may be the case in some samples, in both crops and midguts.

Yes, the reviewer is correct. The FAPROTAX database is assigning these functional categories as present in the communities from both crop and midgut samples. This implies that bacteria that have been identified as parasites or symbionts of animals are present. These relations are dependent on the animal models that are included in the FAPROTAX database. Neither the microeco R package nor the FAPROTAX DB allow us to map the traits or functions to their putative bacterial source. Therefore, inferring which taxa from our dataset could be performing the predicted functions was only done for the 2 categories we found to be significantly different between tissues as pinpointing them to a bacterial source was more straightforward given they could be assigned to exclusive members of the crop community. Identifying animal parasites or symbionts is indeed relevant but it was not in the scope of our study to speculate about them.

Reviewer #1: Line 480-484- It is stated that presence or absence of Elizabethkingia may influence the size of microbiota in the midguts (individual samples). Please clarify in the result sections that samples 4 and 7 (Fig 4) represent the two bottom outliners in (Fig 2).

Text has been added to clarify this in lines 498-500.

Please discuss potential role of Serratia in *Ae. aegypti*.

We mention in the manuscript that *Serratia* was not found, though this is potentially a result of the experimental conditions, strain, and laboratory environment represented. We took the caution of mentioning that other members of the same order (Enterobacterales) were identified. Bacteria from this taxon might have taken the niche and fulfill similar roles in the community given that functional redundancy is possible. We also referred to ASV_22, identified as Enterobacteriaceae, as a possibly unidentified *Serratia* taxonomic unit. We agree a brief mention to the known effects of this commensal in *Ae. aegypti* is relevant and thank the reviewer for the observation.

As suggested by the reviewer we have inserted text indicating the relevance of *Serratia* in *Ae. aegypti* microbiota, particularly as an identified effector of vectorial competence. The text now reads (lines 553-557, and 561-564)

“As recently reviewed by Steven et al (2022) [52], *Serratia* is a relevant member of the microbiota of *Ae. aegypti*. It has been experimentally shown to impact larval survival and vector competence. The mechanisms behind the observed phenotypes are complex and not fully understood given that they may be positive, neutral, or negative depending on the life stage of the host and the pathogen involved in the interactions [52].”

“The fact that neither sequencing nor culture methods revealed the presence of this commensal highlights how spatiotemporal dynamics, and / or strain-specific factors may be at play when we describe the membership of host associated bacterial communities.”

Discuss the differences in the findings between the pooled vs individual samples.

In our original manuscript, we included discussion of differences between individual and pooled samples in terms of bacterial size and discussed how variability was linked with the presence/absence of *Elizabethkingia*. That said, we agree with the reviewer that additional discussion of pooled vs. single samples is warranted. We therefore added text indicating that *Elizabethkingia* presence/absence does indeed correlate with microbiota size (lines 498-500). We also added text indicating that using single vs. pooled samples revealed more inter-individual variation (lines 510-513).

In reporting the bacterial profiles obtained by HTS, we deemed it relevant to: i) test if this variable (sample type) significantly drove the variance captured and ii) test if there was a sample type by tissue type interaction. The PERMANOVA revealed neither was significant, indicating that effect of tissue type was consistent regardless of whether samples were single or pooled. We therefore analyzed beta diversity using all samples together. That said, there are two single midgut samples with clearly different compositions from the other samples and this inter-individual variation is hidden in the pooled samples. We added a statement to reflect this on lines (545-550).

In the introduction:
It will be beneficial to include why the study focused only on the female Ae aegypti rather than both males and females

We acknowledge that there is value in studying both males along with females, but this was beyond the scope of our current study. We chose to study females because most other literature focusing on the gut microbiota of *Ae. aegypti* also focus on the females. This is because females are vectors for human pathogens and one of the main goals of studying the microbiota in this species is to attempt to mitigate the human disease risk associated with this species (e.g. by altering the microbiota to make the mosquito refractory to human pathogen infection). We added the word “female” twice in the introduction to make it clear to the reader that it is the female that transmits pathogens and is therefore of interest (lines 80, 82)

And a figure/drawing of mosquito’s gut anatomy.

We recognize the value of a figure depicting the compartments from which the data were collected. Nonetheless, we think the mosquito digestive tract has been well-depicted in key references we cite in the manuscript when referring to their functions as well as bacteria identified as associated with them. The relevant citations are listed below. We appreciate the reviewer’s suggestion, but we consider it is not necessary given that there are alternative sources readily available.

Gusmão DS, Santos AV, Marini DC, Russo Ede S, Peixoto AM, Bacci Júnior M, Berbert-Molina MA, Lemos FJ. First isolation of microorganisms from the gut diverticulum of Aedes aegypti (Diptera: Culicidae): new perspectives for an insect-bacteria association. Mem Inst Oswaldo Cruz. 2007 Dec;102(8):919-24. doi: 10.1590/s0074-02762007000800005. PMID: 18209929.

Scolari F, Casiraghi M, Bonizzoni M. Aedes spp. and Their Microbiota: A Review. Front Microbiol. 2019 Sep 4;10:2036. doi: 10.3389/fmicb.2019.02036. PMID: 31551973; PMCID: PMC6738348.

Nepomuceno DB, Santos VC, Araújo RN, Pereira MH, Sant'Anna MR, Moreira LA, Gontijo NF. pH control in the midgut of Aedes aegypti under different nutritional conditions. J Exp Biol. 2017 Sep 15;220(Pt 18):3355-3362. doi: 10.1242/jeb.158956. PMID: 28931720.

For clarity, please state whether the mosquitoes are lab reared or field samples when referring to other studies.

Text has been added to clarify this (lines 104, 107, and 567)

**Reviewer #2**

**Specific comments**

-No control of larval experiment

We acknowledge that there was no control of the bacteria introduced to the larvae in our experiment. Our intention was to simulate normal rearing conditions of laboratory colonies of *Ae. aegypti,* and therefore, we did not feel it was appropriate to artificially alter or control the environmental bacterial microbiota that the mosquitoes are exposed to as larvae (as well as the other stages). Our expectation is that the larvae would be exposed to bacteria that are common in a laboratory setting. We did, however, control diet and environmental conditions during larval rearing. We included a statement acknowledging this caveat on lines (127-128)

-No newly emerged mosquitoes

We acknowledge that this was not part of our experimental design. We would predict that the crop and midgut microbiota would change throughout the lifespan of the adult mosquito, particularly after sugar/sucrose feeding or blood feeding. Therefore, we see value in performing a time course experiment looking at the microbiomes at different time points in the adult mosquitoes’ lifespans, but this was outside of the scope of our experiment. Our snapshot shows the state of the microbiome 4-6 days after eclosion and after the gut has been exposed to 10% sucrose meals.

-Supplementary figure 1.I would like to see the read counts rather than the frequency. What does 4, 6, 8, 10 and 12 represent in the x axis? You imply it is reads per samples, does it mean that the samples had only 4, 6, 8, 10 and 12 reads?

The figure is a histogram of read counts per sample. Reads were transformed to a natural logarithm scale along the X axis. (axis label: Ln reads per sample). The Y axis represents the number of samples that have that number of reads. For additional clarity, we modified the figure title and the Y axis label to “Distribution of reads (natural logarithm) per sample” and “Frequency: number of samples” respectively, to make it clearer. The modified figure has been attached to the reviewed version of the manuscript.

-Supplementary figure 2: the shapes are so tiny, I can hardly see them. Please, make them larger.

The figure has been modified as suggested by the reviewer in order to make it clearer.

-Line2 224- 225: “…then scaled reads per sample based on the smallest read count as suggested by [51] in order to preserve proportions.” What was the sequencing depth per sample? What was the smallest and largest read count? You can provide this information here or in the results (see later comments). This information is presented in the supplementary file but it would be good to have it in the results section.

We have provided information in addition to the total number of reads (range of reads, median) in the sequencing overview in the results section of the manuscript (Lines 321-323). Now it reads as follows:

“After buffer blank removal, decontamination and quality filtering~~,~~ the dataset was comprised of 531713 reads, with samples ranging between 9246 to 65275 reads, a median of 37380 reads and 0.80 sparsity. After scaling to...”

-Alpha diversity. I would like to see chao1 values and Shannon diversity index or a justification on why they are not reported.

Our current understanding of bacterial communities associated with mosquitoes (life stages, sex, tissues) states that these communities are low in diversity. Thus, we expected that the communities in both crop and midguts with our model and within our experimental conditions would be composed of few taxa, with dominant members (in terms of read counts). Both Shannon and Simpson indices consider abundance. Nonetheless, the Simpson index is less susceptible to changes in the evenness of rare ASVs and is more appropriate for datasets with dominant members (Hill et al., 2003). In addition, given the nature of Chao1 and Shannon indices, and the fact that we chose to filter rare ASVs through our QC pipeline, the estimation of these indices could be affected.

This is discussed in Daly et al., 2018: “*note that the estimation of the Shannon index is subject to more bias than the estimation of Simpson’s index, since the former is more sensitive to richness, i.e., to the number of species that are sampled*” / “*The Chao estimators estimate the number of unobserved species based on the number of species that are observed either once or twice*.”

We acknowledge the value of other alpha diversity metrics and appreciate the reviewer’s suggestion. However, since we know that our communities are uneven structures with dominant members, we decided to analyze our data and elaborate our discussion based on a single alpha diversity index, in this case one that is readily understandable, not affected by coverage, and reflective of the nature of this assembly.

Hill TCJ, Walsh KA, Harris JA, Moffett BF. Using ecological diversity measures with bacterial communities. FEMS Microbiol Ecol. 2003 Feb;43(1):1–11.

Daly AJ, Baetens JM, De Baets B. Ecological diversity: measuring the unmeasurable. Mathematics. 2018 Jul 10;6(7):119.

-Figure legends: is it necessary to include all this information when it is already stated in the text?

We have attempted to keep our figure legends concise while also providing what we feel are essential details. It is our preference to provide enough details in the figure legends to allow the reader to fully understand the figure without needing to refer to the text. We feel this allows our figures and paper, overall, to be more readily accessible.

-Please include data on the sequencing depth per sample (include minimum and maximum values, mean and SD or SE).

We ask the reviewer to refer to the 3rd answer above this as it is related to the same matter.

- I would also like to see the rarefaction curves to know whether the samples reached saturation or not.

All samples reached saturation except 2. Rarefaction curves are provided now in the Supplementary figure 3 “RarefactionCurves.PNG” and are referred to in the text in line 319.

-Please, provide a figure showing the composition of different bacterial phyla and genera in crop and midgut samples. Any reason why only the family level classification is presented?

The taxonomic family rank has been characterized as more stable than lower ranks when it comes to taxonomic assignation (Mysara et al., 2017 and references therein). Given that the community composition and structure in our samples was expected to be reflective of an uneven assembly, we chose a taxonomic level that reflected the differences between tissues and sample types in both richness and abundance terms. A plot at a higher taxonomic rank (e.g. Phyla) would be informative yet not needed. We agree that a genera or species plot would be valuable, however, these are not stable nor fully reliable as they may be biased depending on the database used to assign IDs to the ASVs. Thus, we believe that a single stacked barplot can provide the reader with the information required to observe the general trends of community structure in the samples, that are then tested in the beta diversity analyses. We ask the reviewer to consider that our intent is to be succinct with both tables and figures while still providing as much useful information to the reader as possible.

-Mysara M, Vandamme P, Props R, Kerckhof FM, Leys N, Boon N, Raes J, Monsieurs P. Reconciliation between operational taxonomic units and species boundaries. FEMS microbiology ecology. 2017 Apr 1;93(4).

-Figure 3: how can you explain the huge difference between pooled and single midgut samples? Could it have been contamination from the crop? How certain are you that was transfer from the crop?

The most likely explanation for why pooled and single midgut samples differ from each other is that we can better detect inter-individual variation among single samples. In samples 2 and 5, *Elizabethkingia* is absent, which appears to primarily be driving the difference between the pooled and single samples. *Elizabethkingia* grows to very high numbers in the midgut when it is present, but it was not present in these two samples, and this presumably allowed for other microbes to colonize and proliferate. As we discuss in the manuscript, several factors may influence the structure of a community: host physiology, bacteria-bacteria interactions, stochasticity and the temporal scale at which each individual was sampled. This holds true to individuals in a population or cohort, and between tissue in an organism. The fact that two of our samples divert from the dominant profile (*Elizabethkingia* driven) reflects how this individual variability can manifest. We added text on lines (545-550) to better acknowledge this issue and address how it affects interpretation of the data.

We also note that, while there were differences between single and pooled midgut samples, the PERMANOVA test confirmed that the sample type (pool vs single) was not a significant driver of variances measured among the samples. Furthermore, there was no significant interaction between this variable and tissue type. Thus, we can confirm that the beta diversity as measured answered our main biological question. It was relevant to process both types of samples (pools and singles) as both allowed us to infer the overall differences between crop and midgut bacterial profiles within our experimental conditions. Nonetheless, we recognize the value and statistical power a larger dataset would bring, and the value of not pooling samples whenever feasible.

Lines 363-371: I would like to see results of Shannon diversity index (which accounts for species abundance) and Chao1.

This question has been answered above.

-Figure 3: Please, increase the size of the shapes. They are too small.

We infer that the reviewer may be referring to Figure 6? As with the Supplementary Figure 2 we modified the size of the elements plotted to make it visually clearer.

- Lines 395-397: “The communities were dominated by reads that map to the genera *Elizabethkingia* (Family: Weeksellaceae), *Tanticharoenia* (Family: Acetobacteraceae), and/or *Asaia* (Family: Acetobacteraceae)”. Figure 3 presents data at family level. Please, present the results at genus level.

As mentioned above, we aimed to provide a figure that allowed the reader to observe the general trend of community structures between the tissues and sample types. We chose the family level as it revealed the trend and is a stable taxonomic rank to work with and report (contrary to genus). We opted to report only the dominant ASVs at the genus level as they are subjects in our discussion. These particular ASVs are addressed at this taxonomic level since they were detected via culture dependent and independent methods and are well known in the literature as members of mosquito microbiota. We don’t consider a stacked barplot at the g-ASV level is required for the reader to understand the nature of the studied bacterial community structures at the position where the figure is located within the manuscript.

-Lines 448-449: “….five functional categories were only found in midguts, including manganese oxidation.” Please, list the other four, they are not easy to spot from the figure. It would also be good to list the 15 that were shared.

The other 4 functional categories only found in midguts are: anoxygenic_photoautotrophy, anoxygenic_photoautotrophy_S_oxidizing, photoautrotophy and phototrophy. They have been added to the text in lines 463-465 as follows:

“Five functional categories were only found in midguts: anoxygenic photoautotrophy, anoxygenic photoautotrophy S oxidizing, manganese oxidation, photoautrotophy, and phototrophy.”

We also listed the 15 shared functions in the text, lines 456-459, as follows:

“aerobic chemoheterotrophy, anaerobic chemoheterotrphy, animal parasites or symbionts, aromatic compound degradation, chemoheterotrophy, fermentation, human associated, human gut, human pathogens all, mammal gut, nitrate reduction, nitrate respiration, nitrogen fixation, nitrogen respiration, and ureolysis.”

Given the inclusion of the shared and midgut exclusive functions, we opted to list the 14 crop exclusive functions too. They are listed in lines 459-463 as follows:

“aliphatic non methane hydrocarbon degradation, chitinolysis, dark hydrogen oxidation, denitrification, human pathogens pneumonia, hydrocarbon degradation, knallgas bacteria, methanol oxidation, methanotrophy, methylotrophy, nitrate denitrification, nitrite denitrification, nitrite respiration, nitrous oxide denitrification.”
